# A cost analysis of the diagnosis and treatment of malaria at public health facilities and communities in three districts in Rwanda

**DOI:** 10.1186/s12936-022-04158-x

**Published:** 2022-05-15

**Authors:** Ornella Masimbi, Janna M. Schurer, Ellen Rafferty, Jean D’ Amour Ndahimana, J. Hellen Amuguni

**Affiliations:** 1 Educational Development and Quality Center, University of Global Health Equity, Kigali, Rwanda; 2 Center for One Health, University of Global Health Equity, Kigali, Rwanda; 3 Department of Infectious Disease and Global Health, Cummings School of Veterinary Medicine at Tufts University, North Grafton, MA USA; 4 Faculty of Nursing, University of Alberta, Edmonton, AB Canada; 5 Department of Infectious Diseases, Partners In Health, Kigali, Rwanda

**Keywords:** Malaria, Community Health Workers, Healthcare costs, Home-Based Management, Rwanda

## Abstract

**Background:**

Malaria is a potentially fatal disease spread by the bites of *Plasmodium*-infected *Anopheles* mosquitoes. Despite long-term efforts to control malaria in Rwanda, malaria incidence increased from 48 to 403 cases/1000 individuals between 2012 and 2016. The diagnosis and treatment of malaria occurs at multiple levels, but the costs of these activities are not well understood. This research was conducted to estimate the direct medical costs incurred by the Ministry of Health in diagnosing and treating malaria in three districts of Rwanda in 2018.

**Methods:**

A cross-sectional and retrospective costing analysis was conducted in three districts that represented low (5–200 cases per 1000 individuals), moderate (> 200–400 cases per 1000 individuals), and high (> 400 cases per 1000 individuals) endemicity regions. Data on malaria cases managed at three healthcare levels (community, health centre, district hospital) was obtained from national databases. The direct medical costs of cases per malaria severity (‘simple malaria’, ‘simple malaria with minor digestive symptoms’, and ‘severe malaria’) were calculated based on the minimum package of health services provided. Total costs for each of the three districts were also calculated.

**Results:**

A total of 298,381 malaria cases were recorded in Burera, Kirehe, and Southern Kayonza districts in 2018. The average unit cost per case ranged from USD 1.36 (for simple malaria at the community level) to USD 92.80 (for severe malaria with cerebral complications at district hospitals). Simple malaria cases managed at health centres and district hospitals were more than two-fold (USD 2.99–USD 3.00) and more than eight-fold (USD 12.10–USD 12.12) higher, respectively, than those managed in the community (USD 1.36). Overall, the Ministry of Health incurred USD 645,647.68 in direct medical costs related to malaria management across the three districts in 2018. Changes in disease rates from different endemicity regions and costs of anti-malarial oral medications significantly impacted the study results.

**Conclusion:**

In Rwanda, severe malaria results in much higher expenses compared to other malaria types. Prompt diagnosis and appropriate treatment are crucial to prevent the progression of simple malaria to severe malaria, to reduce Ministry of Health malaria expenditures, and to reduce community transmission.

## Background

Malaria is a complex and deadly disease, caused by *Plasmodium* parasites transmitted through the bite of infected female *Anopheles* mosquitoes, with *Plasmodium falciparum* responsible for the majority of severe cases in Africa [1, 2]. Malaria is a serious global health issue, with nearly half of the world’s population (3.7 billion) at risk of infection [3, 4]. Of the 228 million malaria cases and 405,000 malaria deaths reported worldwide in 2018, 93% and 94% occurred in Africa, respectively [4]. In Rwanda, the entire population is at risk of malaria, with 19 of 30 districts classified as high endemic zones and the remaining 11 districts classified as endemic [5, 6]. Between 2012 and 2016, Rwanda reported an increase in malaria incidence, from 48 cases per 1000 individuals to 403 cases per 1000 individuals, and an increase in mortality from 419 in 2013 to 715 deaths in 2016 [6].

The World Health Organization (WHO) classifies malaria into two clinical forms, namely uncomplicated malaria, and severe malaria with serious complications [2, 4]. However, to ensure a common understanding of these forms and their clinical management by health professionals, the Rwanda Ministry of Health (MOH) adopted the following terminologies: (1) Simple Malaria (SM), (2) Simple Malaria with Minor Digestive Symptoms (SMD), and (3) Severe Malaria (SVM) [7]. SM clinically manifests with fever, headache, chills, weakness, joint pain, and loss of appetite; SMD cases can also exhibit vomiting and/or diarrhoea. SVM presents with signs of vital distress such as impaired consciousness, convulsions, hypoglycaemia, severe anaemia, respiratory distress, and renal impairment [7–9]. The MOH terminologies were used throughout this project.

Early diagnosis and prompt treatment of malaria reduce the number of severe cases and deaths [10, 11]. In Rwanda, malaria is primarily managed by Community Health Workers (CHWs), members of local villages with no formal medical training who are trained to provide basic health services in their communities. CHWs are motivated through performance-based incentives for the health services delivered [7, 12]. Through the Home-Based Management (HBM) programme, CHWs diagnose and treat SM cases in villages, and refer complicated cases to nearby health centres. Patients referred to health centres receive primary curative consultations from nurses while those attending district hospitals are primarily consulted by general practitioners [13, 14]. The national guidelines for the treatment of malaria require laboratory confirmation of infection before initiating any malaria-specific treatment. Blood smear is the standard diagnostic test.

Rapid Diagnostic Tests (RDTs), on the other hand, are used by CHWs in villages but can also be used at healthcare facilities during weekends or in emergency cases when laboratory technicians are not available [7]. The national guidelines also recommend artemisinin-based combination therapy (ACT) as the first-line of treatment of SM cases through HBM or at any healthcare facility while artesunate is used as the first-line drug for treating SMD and SVM. Patients diagnosed with SVM at health centres must be referred to the nearest district hospital for further management [7]. Although preventable and curable, malaria remains an important challenge to the public health sector, contributing to the poverty of people and households, and limiting socio-economic development [1]. For the last two decades, malaria management has cost up to USD 300 million each year in sub-Saharan Africa and reduced the Gross Domestic Products (GDP) by 1.3% [3, 15]. At the household level, families incur direct medical costs (e.g., diagnosis, malaria drugs, consultation), direct non-medical costs (e.g., travel costs, food on the way to a health facility); and indirect costs (e.g., lost wages due to illness or caregiving activities) [15, 16]. These costs can trap households in a cycle of illness, suffering, and poverty that restrict individuals from seeking and utilizing healthcare [5, 16, 17]. To help Rwandan citizens access medical care at an affordable cost, the Government of Rwanda introduced Community Based Health Insurance (CBHI), a national public insurance system. Members of CBHI pay 200Frw for medical care received at health centres, while at district hospitals, provincial or referral hospitals, they pay 10% of the total cost [18]. The high incidence of malaria cases also consumes considerable resources in health facilities [19].

In 2016, WHO set a goal to reduce the global malaria case incidence by 90% by the year 2030 [20]. Characterizing the costs of diagnosis and treatment is a critical step towards achieving this goal as it informs decision-making about the provision of appropriate funding for malaria management and prevention, potentially lowering costs for patients and providers [19]. However, this information is not publicly available in Rwanda. Thus, the aim of this study was to estimate the direct medical costs incurred by the MOH in diagnosing and treating malaria in three districts of Rwanda in 2018.

## Methods

### Study population and data sources

Rwanda is a landlocked country located in the Great Lakes region of Eastern Africa bordered by Burundi, the Democratic Republic of Congo, Uganda, and the United Republic of Tanzania. The Rwandan population is estimated at 12.95 million people, making it one of the most densely populated countries in Africa [14, 21]. The GDP per capita in Rwanda was USD 826.30 in 2018 [22].

The health system in Rwanda is led by the MOH, which regulates all health programmes in the country. Health services are provided through public, private, faith-based, and non-governmental sectors. The public sector is operated at three different levels: the central level with the MOH on top and the referral hospitals it manages; the intermediate level composed of provincial hospitals and district hospitals; and the peripheral level consisting of health centres, health posts, and CHWs in the community [23]. The public health sector is financed by the Government of Rwanda and external donors. The malaria control programme is primarily financed by the Government of Rwanda but relies on funds from the Global Fund and the President’s Malaria Initiative [24].

**Fig. 1:**
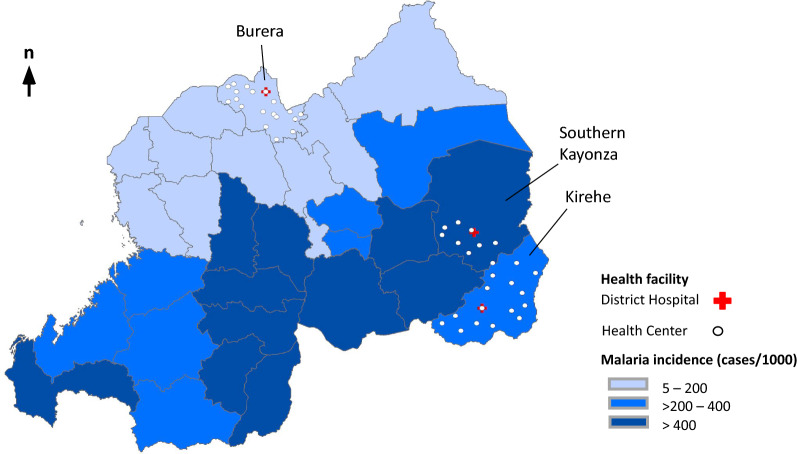
Malaria incidence in health facilities under joint Ministry of Health and PIH management in Rwanda, 2018

A retrospective, cross-sectional study was conducted to estimate the direct medical costs for malaria across communities, health centres and district hospitals in Burera, Kirehe and Southern Kayonza districts (Fig. 1). These study districts were chosen based on the malaria incidence, with Burera classified as very low (5–200 cases per 1000 individuals), Kirehe as moderate (> 200–400 cases per 1000 individuals), and Kayonza as high (> 400 cases per 1000 individuals) [13]. Healthcare in the target districts is managed and financed by the MOH in collaboration with Partners In Health (PIH), a non-profit organization founded in 1987 with the mission to provide a preferential option for poor and underserved communities in healthcare. PIH supports the provision of quality care to more than 860,000 individuals through a network of approximately 6,400 CHWs, 43 health centres, and three district hospitals [25]. This is possible through PIH’s enhancement of CHW networks and nursing supervision; and its support in service delivery, increasing human resources, and implementing a monitoring and evaluation system in those facilities. Besides this, PIH also provides food, agriculture tools, training; housing, and economic support to the poorest families in Kirehe, Burera, and Kayonza villages [26]. Age-specific primary data on hospitalized and non-hospitalized malaria cases and deaths from January to December 2018 was extracted from the Rwanda Integrated Health Management Information System (HMIS), an electronic database storing monthly counts of health centre visits, district hospital admissions, and deaths at all health facilities. The data on malaria cases treated by CHWs was obtained from System Information Community Data (SISCOM,) a dataset containing community diagnosis, treatment, essential drug logistics, and mortality information [27, 28].

Included in this study were (i) SISCOM records of positive SM cases (ii) HMIS records of positive SM and SMD cases treated at health centres (iii) HMIS records of positive SM, SMD, and SVM cases treated at district hospitals.

The population size estimates in all the 30 districts of Rwanda were obtained from the 2012 population and housing census in Rwanda, and these values were inflated to the 2018 values using the National Institute of Statistics of Rwanda [29]. Malaria endemicity rates in the rest of the country were also extracted from government official documents [13]. These allowed the categorization of all 30 districts according to their endemicity rate with 11 districts grouped under low endemicity area; and eight districts and 11 districts grouped under moderate and high endemicity areas, respectively.

### Cost estimation

A costing analysis was conducted from the perspective of the MOH. Unit prices of ACT, artesunate, and RDTs were collected from the “Malaria, Neglected Tropical Diseases, and Other Parasitic Diseases Division” responsible for the prevention, vector control, and case management of malaria in Rwanda. The unit prices of other pharmaceutical products, medical supplies, and health services (consultation, medical visits, laboratory tests) dispensed at health facilities were obtained from district pharmacies operated by the MOH in the three districts. Cost estimates were generated in Rwandan francs and then converted to US dollars using the National Bank 2018 exchange rate [30].

Direct medical costs were estimated per malaria type at each of the three levels of medical care. These costs were based on the minimum package of essential clinical services to which all suspected malaria cases can access at the community level (rapid testing, anti-malarial drugs); and at health centre and district hospital levels (consultation, laboratory testing, anti-malarial drugs, other treatment and/or inpatient care; Table 1). Cost estimations were based on the first line of malaria pharmaceutical treatment recommended by the national protocol. For pregnant women, this protocol recommends oral quinine as the treatment of SM during the first trimester, and ACT during the second and third trimesters [7]. However, 99% of pregnant women in Rwanda start their antenatal care visits in the second trimester of pregnancy at a median gestational age of four months [14]. Therefore, pregnant cases were assumed to be in their second trimester of pregnancy where they were prescribed ACT for SM.

**Table 1:** Malaria diagnosis and treatment services provided across public health facilities in Rwanda

	Home-based management	Health centre	District hospital
SM^a^			
Diagnosis	RDT^d^	Consultation (nurse) Blood smear and/or RDT^d^	Consultation (GP^k^) Blood smear Complementary lab exam (CBC^l^)
Treatment (first line)	ACT^e^	ACT^e^ Supportive treatment^f^ (antipyretics)	ACT^e^ Supportive treatment^f^ (antipyretics)
SMD^b^			
Diagnosis	N/A	Consultation (nurse) Blood smear and/or RDT^d^ Complementary lab exam (Haemoglobin)	Consultation (GP^k^) Blood smear Complementary lab exam (CBC^l^)
Treatment (first line)	N/A	Hospitalization^g^ (LOS:2)^h^ Artesunate & Supportive treatment^f^ (antipyretics, ORS^i^, IV^j^ fluids) ACT^e^ on discharge	Hospitalization^g^ (LOS:2)^h^ Medical visit in hospitalization Artesunate Supportive treatment^f^ (antipyretics, IV^j^ fluids) ACT^e^ on discharge
SVM^c^			
Diagnosis	N/A	Consultation by a nurse before transfer	Consultation (GP^k^) Blood smear Complementary lab exam (CBC^l^, blood glucose test, urea, creatinine)
Treatment (first line)	N/A	–	Hospitalization^g^ (LOS:5)^h^ Medical visit by (GP^k^) Artesunate & ACT^e^ on discharge If cerebral form (added treatment): Anticonvulsants, antipyretics, antibiotics IV^j^ fluids and medical supplies (e.g., NG^n^ tube, Foley catheter)

^a^ SM: Simple Malaria; ^b^SMD: Simple Malaria with Minor Digestive Symptoms; ^c^SVM: Severe Malaria; ^d^RDT: Rapid Diagnostic Test; ^e^ACT: Artemisinin-based combination therapy; ^f^ Supportive treatment: treatment given to relieve symptoms or prevent further complications; ^g^Hospitalization: cost of in-patient stay per day; ^h^LOS: Length of in-hospital stay in days; ^i^ORS: Oral Rehydration Salt; ^j^IV: Intravenous, ^k^GP: General Practitioner; ^l^CBC: Complete Blood Count; ^n^NG: Nasogastric

### Pricing and cost measurement

The direct medical costs per malaria type at the three levels of medical care included the costs of malaria commodities (RDTs, anti-malarial drugs), and other clinical services (consultation, hospitalization, medical visit, additional laboratory tests, medications, and medical supplies). Clinical services varied according to the severity of the patient’s symptoms. The cost of each healthcare activity (e.g., diagnostic test, anti-malarial drugs) was calculated using its unit price and consumption quantities (e.g., number of RDTs). While the unit prices of malaria commodities, consultation, hospitalization, and additional laboratory tests did not vary across the three districts, the unit prices of other prescribed medications differed due to additional transportation and storage costs. Moreover, HMIS and SISCOM only reported the consumption quantities of malaria commodities; therefore, assumptions were made about the care SM, SMD, and SVM patients would receive based on HMIS & SISCOM data availability and variations in unit prices. Three scenarios were used to estimate the average cost per each healthcare activity per healthcare level (HBM, health centre, or district hospital); the terms “reported consumption quantities” were used to refer to the quantities of malaria commodities and blood smears reported under HMIS and SISCOM, while the terms “unreported consumption quantities” referred to the estimated quantities of other health services whose consumption quantities were initially not reported by the two databases:

A. For the reported consumption quantities, the average cost of RDT or blood smear, or anti-malarial drugs at the three healthcare levels was calculated as such:
  $$\begin{aligned} \,Average\;cost\;per\;health\;activity\;by\;healthcare\;level: \\ & = \frac{{unit\;price\;of\;healthcare\;activity \times consumption\;quantities\;by\;healthcare\;level}}{{Total\;malaria\;cases\;by\;healthcare\;level}} \\ \end{aligned}$$

B. For the unreported consumptions quantities, the variations in unit prices were referred to in the estimations as follows:
1. If the unit price did not vary across the three districts, the following formula was used:
o * Average cost per health activity by healthcare level = unit price × frequency recommended by the national protocol per malaria case type*
2. If unit price varied across the three districts, the minimum cost (based on lowest unit price) and maximum costs (based on highest unit price) were estimated as follows:
o ***Minimum average cost per case* = ***lowest unit price* × *recommended dosage* × *estimated number treatments*
o ***Maximum average cost per case*** = *highest unit price* × *recommended dosage* × *estimated number treatments*

Based on the different healthcare activities conducted in the management of each malaria type (Table 1), the average costs of each healthcare activity were summed to get the average unit cost per malaria case type by healthcare level. Specifically, in HBM, since RDT and ACT are the only healthcare activities used for managing SM cases, their average costs were added together to get the average cost per SM case in HBM (Table 2: Eq. 1).

**Table 2:** Equations used to calculate minimum and maximum average unit costs and total costs per malaria type

**Equation 1**	***Average cost per SM***^***a***^*** case in HBM***^***b***^ = *Diagnostic cost* + *Antimalarial drugs cost*
Equation 2	***Minimum average cost per malaria case type by healthcare level*** = *Consultation cost*_*t,f*_ + *Medical* *visit* *cost*_*t,f*_ + *Diagnosis* *cost*_*t,f*_ + *Antimalarial* *drugs* *cost*_*t,f*_ + *additional laboratory tests costs*_*t,f*_ + *other prescribed medications minimum costs*_*t,f*_
Equation 3	***Maximum average cost per SM***^***a***^*** or SMD***^***c***^*** case by healthcare level*** = *Consultation cost*_*t’,f*_ + *Medical visit cost*_*t’,f*_ + *Diagnosis cost*_*t’,f*_ + *Antimalarial drugs cost*_*t’,f*_ + *additional laboratory tests costs*_*t’,f*_ + *other prescribed medications maximum cost*_*t’,f*_
Equation 4	***Maximum average cost per SVM***^***d***^*** case at DH***^***e***^** = ***Equation 2* + *other prescribed medications maximum cost* + *medical supplies costs*
Equation 5	***95% Confidence Interval of a count variable* = ***C* ± *Z*_1*–*α/2_*√C*
Equation 6	***Minimum total cost per malaria type by health facility*** = *minimum average unit cost *_*t, l*_*** x**** lower bound 95% CI cases *_*t, l*_
Equation 7	***Maximum total cost per malaria type by health facility*** = *maximum average unit cost*_*t, l*_*** x**** higher bound 95% CI cases*_*t, l*_
Equation 8	***Total costs of diagnosing and treating malaria in the three districts:*** *Minimum: sum of minimum total cost per malaria type* *Maximum: sum of maximum total cost per malaria type*

^a^SM: Simple Malaria; ^b^HBM: Home-Based Management; ^c^SMD: Simple Malaria with Minor Digestive Symptoms; ^d^SVM: Severe Malaria, ^e^District Hospital; t applies to severity of malaria (SM, SMD, SVM); f applies to health facility (health centre, district hospital); t’ only applies to SM or SMD; *l* is the healthcare level (HBM, health centre, district hospital)

Furthermore, for health facilities where additional medications were given in addition to antimalarials, the minimum and maximum average cost of these drugs were added to the other provided services in order to get a minimum (Table 2: Eq. 2) and maximum average unit cost per malaria case by type and facility (Table 2: Eq. 3). All SVM cases were assumed to have presented as cerebral malaria, as it is the most severe presentation and the most common cause of death in patients with malaria [31]. Thus, for severe cases, the minimum average cost per SVM case was estimated based on the minimum health package for SVM (accounting only for malaria-related drug and supply costs; Table 2: Eq. 2). In comparison, the maximum average unit cost per SVM case was obtained by taking the minimum cost for SVM (Table 2: Eq. 2) and adding the average costs of additional medications and supplies used in the management of cerebral malaria (Table 2: Eq. 4).

### Statistical analysis

Pearson's Chi-square test was used to test for associations between demographic variables or malaria type and health facility type (health centre or district hospital). P-values of less than 0.05 were considered statistically significant. In comparison to the HMIS database which collected four demographic characteristics (age, sex, pregnancy status, and malaria severity), the SISCOM database only reported one demographic characteristic (age) of malaria cases managed in the community. This was categorized into two age groups: 6–59 months and > 5 years old.

### Total annual cost estimates

Based on the number of SM, SMD, and SVM cases reported in the HMIS and SISCOM data, a Poisson distribution was used to compute a 95% confidence interval (CI; Table 2: Eq. 5) around malaria cases reported under HBM, health centre, and district hospital. The total costs by malaria type by healthcare level were calculated using the lower and upper bounds of the 95% CI, along with the minimum and maximum average cost per case. Each minimum average unit cost per case was multiplied by the minimal count of cases by malaria case type (lower bound—95% CI) to get a minimum total cost per malaria case type by different healthcare levels (Table 2: Eq. 6). Similarly, the maximum average unit cost per case was multiplied by the maximum count of cases (higher bound—95% CI) to calculate the maximum total cost per malaria case type by different healthcare levels (Table 2: Eq. 7). All minimum total costs per malaria type were summed up to get the lowest value for the total annual healthcare costs of diagnosing and treating malaria in the three districts. The highest value for total costs was equal to the sum of all maximum total costs per malaria type (Table 2: Eq. 8).

### Sensitivity analysis

One-way sensitivity analyses were conducted on variables whose estimates were uncertain or prone to change over time. These included the costs of RDT, blood smear (at health centres and districts hospitals), ACT (treatment dosages for adults and children weighing less than 15 kg), artesunate, the hospitalization at health centres and at district hospital levels.

A sensitivity analysis on malaria incidence was also carried out by varying the disease rate between low, moderate, and high endemicity areas. Disease rates for each malaria severity (SM, SMD, SVM) were obtained by applying the number of cases in each district over the total population in that specific district, these rates were presented in percentage (Table 3). The disease rates in Burera were used as the rates for a low endemicity area, and the rates in Kirehe and Kayonza were used for moderate and high endemicity areas, respectively. The rates in each district were then applied to the total population of each of the other two districts in different endemicity areas (e.g., rate from Burera applied to the total population in Kirehe and in Kayonza, and vice versa) to get the number of malaria cases if all three districts had low, moderate, and high rates of malaria.

**Table 3:** Population estimates and disease rates

Parameters	Burera	Kirehe	Kayonza	Source
Population estimate (2018):	388,323	392,691	397,063	[29]
Disease rate (% of total) per healthcare level				
Simple Malaria:				
Home-base management	0.079	5.871	44.714	[29, 32]
Health centre	1.136	4.157	18.671	
District hospital	0.001	0.009	0.085	
Simple Malaria with Minor Digestive Symptoms:				
Health centre	0.012	0.036	0.274	[29, 32]
District hospital	0.006	0.013	0.101	
Severe Malaria:				
District hospital	0.005	0.018	0.098	[29, 32]

Furthermore, the total direct medical costs of diagnosing and treating malaria across the country were estimated by using these disease rates as archetypes for districts with similar endemicity. These rates were applied to the total population in each district of the same category to get the malaria cases in each district. The average costs per case were then applied to total estimated malaria cases to determine the total costs of diagnosing and treating malaria in all 30 districts of Rwanda in 2018.

For the other inputs, a low and high case scenario analysis was conducted by varying their unit costs with low and high estimates retrieved from governmental documents and peer-reviewed literature (Table 4). This analysis yielded the lowest and highest estimates of malaria impact on the overall direct medical costs incurred by the MOH in the three districts in 2018.

**Table 4:** Healthcare seeking behavior and their unit costs (USD)

Parameters	Base unit cost	Low estimate	High estimate	Source
Consultation				
By a nurse	1.18	-	-	[33]
By a GP^a^:	4.26	-	-	[34]
Hospitalization in ward/day^1^:				
At health centre:	0.71	0.51	7.7	[33‚ 35]
At district hospital:	1.89	1.37	11.7	[34, 36]
Inpatient medical visit (GP^a^):	1.18	-	-	[34]
Diagnostic test:				
RDT^b,1^	0.65	0.57	1.1	[37–39]
Blood smear^1^				
At health centre:	1.07	0.77	1.53	[33, 40]
At district hospital	1.50	1.44	1.91	[34‚ 36, 41]
Haemoglobin	0.93	-	-	[34] [34] [34] [34] [34]
Complete blood count	5.91	-	-	
Urea	5.32	-	-	
Creatinine	5.32	-	-	
Glycaemia	3.99	-	-	
Drug costs				
ACT^c,1^				
6 × 1 (package size: 6 tablets)^d^	0.55	0.36	2.9	[37‚ 38, 42]
6 × 2 (package size: 12 tablets)^e^	0.49	-	-	[37] [37]
6 × 3 (package size: 18 tablets)^f^	0.45	-	-	
6 × 4 (package size: 24 tablets)^g^	0.58	0.5	2.9	[37, 38, 42]
Artesunate^1^:	1.72	1.06	2.76	[37, 43]
Antipyretic:				
Oral tablet	0.01	-	-	District pharmacies
Syrup	0.44	-	-	
Anticonvulsant:	0.78	-	-	
Antibiotic:				
Ampicillin	0.29	-	-	District pharmacies
Chloramphenicol	0.77	-	-	
Oral Rehydration Salt:	0.11	-	-	
Intravenous fluids:	0.62	-	-	
Medical supplies				
Foley catheter	0.61	-	-	
Nasogastric tube	0.19	-	-	

^a^GP: General practitioner; ^b^RDT: Rapid diagnostic test; ^c^ACT; Artemisinin-based combination therapy; ^d^ACT (6 × 1): dosage for children with 5 to < 15 kg body weight; ^e^(6 × 2): for children with 15 kg to < 25 kg body weight; ^f^(6 × 3): for children with 25 kg to < 35 kg body weight; ^g^(6 × 4): for adults and children with > 35 kg; ^1^input used in sensitivity analysis

## Results

### Study population and malaria cases

A total of 298,381 positive malaria cases were diagnosed and treated in Kirehe, Kayonza, and Burera districts in 2018. Of these, 97,479 cases were reported by health facilities with 96,145 (98.6%) cases managed at health centres and 1,334 (1.4%) cases managed at district hospitals. Most were SM (95,249 cases), followed by SMD (1,750 cases) and SVM (480 cases). Southern Kayonza accounted for most cases (76,349 cases), followed by the Kirehe (16,627 cases) and Burera (4503 cases; Table 5). Although gender was not reported for 9,149 cases, those remaining were evenly distributed among males and females. Most female patients were not pregnant (45,892 cases). Age, pregnancy status, malaria type, and district were statistically different between the two health facilities (p < 0.001).

**Table 5:** Demographic characteristics of malaria cases reported in HMIS by health facilities

	Health centre [N = 96,145 (98.6)]	District hospital [N = 1,334 (1.4)]	Total (N = 97,479)	Pearson X^2^ p-value
	n (%)			
Age (years)				
< 5	9,150 (96.0)	385 (4.0)	9,535 (100.0)	** < 0.001**
5–19	39,565 (99.2)	309 (0.8)	39,874 (100.0)	
> 20	47,430 (98.7)	640 (1.3)	48,070 (100.0)	
Sex				
Male	39,758 (98.6)	583 (1.4)	40,341 (100.0)	**0.15**
Female	47,238 (98.4)	751 (1.6)	47,989 (100.0)	
Missing^1^	9,149 (100.0)	0 (0.0)	9,149 (100.0)	
Pregnancy status (female patients)				
Pregnant	1,920 (91.6)	177 (8.4)	2097 (100.0)	** < 0.001**
Non-Pregnant	45,318 (98.7)	574 (1.3)	45,892 (100.0)	
Malaria severity				
SM^a^	94,872 (99.6)	377 (0.4)	95,249 (100.0)	** < 0.001**
SMD^b^	1,273 (72.7)	477 (27.3)	1,750 (100.0)	
SVM^c,2^	0 (0.0)	480 (100.0)	480 (100.0)	
District				
Burera	4,455 (98.9)	48 (1.1)	4,503 (100.0)	** < 0.001**
Kirehe	16,467 (99.0)	160 (1.0)	16,627 (100.0)	
Southern Kayonza	75,223 (98.5)	1,126 (1.5)	76,349 (100.0)	

^a^ SM: Simple Malaria; ^b^SMD: Simple Malaria with Minor Digestive Symptoms; ^c^SVM: Severe Malaria

^1^Missing: HMIS did not report the sex of patients less than 5 years who were treated in health centres

^2^SVM cases recorded as being referred to district hospitals from health centres were excluded from the HC section to avoid double-counting

Of the total cases recorded in the three districts, 200,902 SM cases were reported by CHWs in SISCOM. Of these, 177,543 (88.4%) were from Kayonza, 23,053 (11.5%) were from Kirehe, and 306 (0.1%) were from Burera District. Most cases involved individuals more than 5 years old (83.5%; Table 6).

**Table 6:** Characteristics of SM cases managed through HBM (N = 200,902) in three study areas in Rwanda

Demographic characteristic	SM^1^ cases in HBM^2^; n (%) [N = 200,902]
Age group:	
6–59 months	33,172 (16.5)
> 5 years	167,730 (83.5)
District	
Burera	306 (0.1)
Kirehe	23,053 (11.5)
Southern Kayonza	177,543 (88.4)

^1^SM: Simple Malaria, ^2^HBM: Home Based Management

### Cost analysis results

The distribution of the direct medical costs of diagnosing and treating malaria in the three districts is shown in Table 7. The average healthcare cost per episode of SM was USD 1.36 in HBM and USD 3.00 and USD 12.12 at health centres and hospitals, respectively. The cost per episode of SMD at district hospitals was USD 27.38, which was more than double the cost at health centres. The average cost per episode of SVM at district hospitals ranged from USD 86.98 to 92.80.

**Table 7:** Minimum, maximum average unit, and total costs per malaria type treated in the community, at health centres, and district hospitals

	# patients (95% CI^a^) [N = 298,381]	Min avg^b^ cost	Max avg^b^ cost	Total cost/severity/facility type (USD^c^)	
				Min	Max
Simple Malaria					
HBM^d^	200,902 (200,023–201,781)	1.36		272,031.95	274,421.49
Health centre	94,267 (94,268–95,476)	2.99	3.00	281,862.19	286,427.13
District hospital	377 (339–415)	12.10	12.12	4,101.17	5,030.53
Simple Malaria with Minor Digestive Symptoms					
Health centre	1,273 (1,204–1,344)	12.43	12.45	14,966.22	16,732.30
District hospital	477 (434–520)	27.37	27.38	11,883.78	14,232.40
Severe Malaria					
District hospital	480 (437–523)	86.98	92.80	38,015.48	48,528.83
Total costs in 2018:				**622,860.78**	**645,372.68**

^a^CI: Confidence Interval; ^b^Avg: average; ^c^USD: United States Dollar; ^d^ HBM: Home-based Management

Applying the cost per malaria case to the number of malaria cases (based on disease severity) at each facility yielded total costs ranging from USD 4,101.17 to USD 286,427.13 for SM; from USD 11,883.78 to USD 16,732.30 for SMD; from USD 38,015.48 to 48,528.83 for SVM cases. Overall, in 2018, the diagnosis and treatment of malaria in the three districts cost the MOH USD 622,860.78–645,372.68.

### Sensitivity analysis

The results of sensitivity analyses are presented in Table 8. This analysis revealed significant variations in low and high estimate impact on the total direct costs incurred by MOH in diagnosing and treating malaria in the three districts in 2018. Applying the lowest disease rates to all three districts (referring to a low endemicity area) showed a significant drop in the overall costs by up to 11 times (from USD 622,860.78–645,372.68 to USD 52,686–59,182) while referring to disease rates in a high endemicity area increased the overall costs by more than 2 times (USD 1,544,794–1,583,129). Disease rates were the most sensitive parameters, followed by the costs of ACT adult treatment dosage (6 × 4). These effects were also reflected in the overall economic impact analysis, where varying all low and high parameters simultaneously yielded an overall total cost ranging between USD 549,169–1,343,601. Changes in the costs of hospitalization, blood smear, artesunate and ACT paediatric treatment dosage (6 × 1) did not significantly impact the overall costs.

**Table 8:** Sensitivity analysis of uncertain parameters on the overall costs

Baseline total cost (USD^a^)	Min	Max
	622,860.78	645,372.68
Parameter	Low parameters estimate effect (USD^a^)	High parameters estimate effect (USD^a^)
Disease rate:		
Low endemicity area	52,686	59,182
Moderate endemicity area	263,812	277,501
High endemicity area	1,544,794	1,583,129
Hospitalization in the ward:		
At health centre:	622,380	664,128
At district hospital:	622,407	655,617
RDT^b^	606,695	744,050
Blood smear		
At health centre:	597,430	685,993
At district hospital:	622,841	645,545
ACT^c^		
6 × 1^d^	614,174	752,363
6 × 4^e^	609,406	1,026,020
Artesunate	620,485	649,575
Lowest and highest economic impact^1^	549,169	1,343,601

^a^USD: United States Dollar; ^b^RDT: Rapid diagnostic test, ^c^ACT: Artemisinin-based combination therapy; ^c^6 × 1: dosage for children with 5 kg to < 15 kg body weight; ^d^6 × 4: dosage for adult and children with > 35 kg body weight; ^1^Lowest and highest economic impact: overall effect on total costs produced by varying all low cost and high-cost estimates simultaneously

Furthermore, estimating the total direct medical costs incurred by MOH in all 30 districts of Rwanda in 2018, produced an overall total cost ranging between USD 6,597,378 to 6,724,673. High endemic districts accounted for most of the costs (USD 5,640,041–5,728,710) followed by moderate endemicity districts (USD 774,618–USD 800,053) and low endemic districts (USD 182,718.61–195,910.60).

## Discussion

This study provides the total costs, minimum and maximum average costs per malaria case associated with the diagnosis and treatment of malaria in Burera, Kirehe, and Southern Kayonza districts in 2018. The findings of this study indicated that the MOH spent USD 645,372.68 in malaria diagnosis and treatment costs in the target districts in 2018. The estimates from this study also yielded an average cost per case ranging between USD 1.36 to USD 90.66. The overall economic burden of malaria in the three districts was mostly sensitive to disease rates and the costs of ACT. As expected, rates in high endemicity areas were highly sensitive because the number of malaria cases substantially increased in all three districts. Among the treatment costs, the costs of ACT in adult dosage form (6 × 4) were the most sensitive. Similar findings have been reported in high malaria transmission regions in Mozambique where the costs of medical treatment increased with age due to higher dosage needed in adults [44].

By 2030, the WHO aims to achieve ambitious reductions in the global health burden of malaria, including decreasing incidence and fatalities by 90% and eliminating malaria from at least 35 countries [20]. In Rwanda, the MOH strategic plan aims to decrease malaria incidence by 60% and mortality by 40% by 2024 [45]. Per capita, approximately USD 6.06 is spent on various control measures each year, with significant inputs from The President’s Malaria Initiative and the Global Fund [46]. Achieving long-term reductions in malaria burden will require sustained attention to both risk reduction (e.g., integrated vector control) and health systems strengthening (e.g., case management), especially as drivers for malaria resurgence are complex. Altogether, this suggests the need for strategic investments based on current spending, future incidence forecasts, and environmental models to finance national and global goals for malaria control.

In this study, more than half of malaria patients (67.3%) were diagnosed with SM and treated by CHWs through the HBM programme. Treatment of SM at the community level was the least expensive per episode (USD 1.36); SM cases diagnosed and treated in health centres and district hospitals were more than twofold (USD 2.99–3.00) and more than eightfold (USD 12.10–12.12) higher, respectively. These findings indicate that diagnosing and treating SM increased substantially as the level of health facilities increased. This is because the cost of the health services package provided by CHWs is lower than those provided at health centre and district hospitals, mainly due to the absence of unit cost of CHWs consultations in the MOH costing system. The differences in consultation fees at health centres (nurses’ consultations) and at district hospital (GP’s consultations) as well as the costs associated with additional laboratory tests done (haemoglobin, CBC) at those facilities might have contributed to those variations [47]. The above facts might also have contributed to having an average cost per SMD case treated at district hospital higher than at health centre (USD 27.37–27.38 vs USD 12.43–12.45: Table 7). These findings are similar to those in rural Zambia where malaria cases diagnosed and treated through HBM cost less than those managed at health facilities (USD 4.22 versus USD 6.61) [48].

Overall, 0.1% of malaria cases in the three study districts were categorized as severe. When compared to other malaria types managed at the same facility, SVM cases were far the most expensive. This is attributed to longer hospital stays, the use of different forms of anti-malarial drugs (intravenous and tablet form), additional laboratory tests, and medical adjuvants in treating SVM and its neurological sequelae. Comparison of health package expenses showed that consultation expenses were higher in district hospitals due to the type of healthcare provider (physicians versus nurses at health centres). This aligned with a previous evaluation of SVM costs in Ghana, Kenya, and Tanzania [49] and a systematic review of malaria interventions in sub-Saharan Africa, Asia, and South America [50]. The SVM cost estimates from the current study are also similar to those in the Democratic Republic of Congo where direct hospital costs of a SVM case ranged from USD 27 in religious hospitals to USD 139 in state hospitals [51].

Early diagnosis and prompt treatment are crucial for patient prognosis and population-level control. Treating infected individuals reduces the number of reservoirs, decreases transmission to others within a community, and reduces the pressure of resistance to anti-malarial drugs [45, 52]. Moreover, SM can progress to SVM in the absence of prompt and effective treatment within the first 24 h after the onset of fever, especially in children [53–55]. The early symptoms of malaria in children are non-specific and might be easily confused with other conditions, such as viral syndromes or acute gastroenteritis [56]. In addition, parents' daily work duties (e.g., farming), self-treatment with anti-malarial drugs, care-seeking from informal providers (e.g., traditional healers), and long travel distances to health facilities in remote rural areas can delay access to prompt diagnosis and thus exacerbate poor outcomes and high treatment costs [57–59].

In Rwanda, solutions to strengthen malaria detection and response must be cost-effective, suitable for rural and remote regions, and feasible in low-resource settings where CHWs and patients might lack telecommunication and transportation options. Interventions could include scaling up the HBM programme, increasing the number of CHWs assigned to malaria surveillance, providing regular and appropriate training, and ensuring an adequate supply of diagnostic kits and anti-malarial drugs. As seen elsewhere, one advantage of promoting the HBM programme is that patients can access appropriate care without traveling to distant facilities and incurring transport fees [60]. In Burkina Faso, early treatment of malaria cases within the community was demonstrated to reduce the risk of developing SVM by 50% [61].

This study has several limitations. First, the consumption quantities for additional laboratory tests, drugs, and other supplies were not reported under the malaria section in the two databases. Second, the complications associated with malaria (including anaemia, hypoglycaemia, kidney failure) and the variations in the treatment protocol for individual cases (pregnant versus non-pregnant) were not studied. Therefore, the study estimates are likely to underestimate the true costs of malaria management in each district. Although this study had some limitations, the significant reduction in overall costs observed with the disease rates in low endemicity emphasizes the importance of improving malaria prevention through improved access to prevention services and resource allocation to reduce malaria morbidity in Rwanda.

## Conclusions

Malaria is managed at all healthcare levels across Rwanda. This analysis demonstrated that malaria is least expensive when diagnosed and treated by HBM compared to health centres/hospitals and that SVM costs were considerably higher than those of other malaria types. The findings from the current study emphasize the need to support early diagnosis and treatment at the community level to optimize patient outcomes, reduce community transmission, and optimize resource allocation. A comprehensive assessment of malaria control programmes (e.g., integrated vector management), direct medical costs, and economic burden would further support government efforts to optimize resource use and minimize malaria cases.

*Ethical approval and consent to participate.*

This research was reviewed and approved by the Institutional Review Boards at the University of Global Health Equity. Approval to access HMIS and SISCOM data on malaria cases in the three districts was obtained from the Rwanda Biomedical Center.

## Data Availability

The datasets used and/or analysed during the current study are available from the corresponding author on reasonable request.

## Abbreviations

ACT: Artemisinin-based combination therapy
CBC: Complete Blood Count
CHWs: Community Health Workers
DH: District Hospital
GDP: Gross Domestic Product
HBM: Home-Based Management
HMIS: Health Management Information System
IV: Intravenous
MOH: Ministry of Health
NG: Nasogastric
ORS: Oral Rehydration Salt
PIH: Partners In Health
RDT: Rapid Diagnostic Test
SISCOM: System Information Community Data
SM: Simple Malaria
SMD: Simple Malaria with Minor Digestive Symptoms
SVM: Severe Malaria
WHO: World Health Organization
USD: United States Dollars
